# The Orthodontics-Periodontics Challenges in Integrated Treatment: A Comprehensive Review

**DOI:** 10.7759/cureus.38994

**Published:** 2023-05-14

**Authors:** Lujain Alsulaimani, Hashim Alqarni, Mohammed Akel, Fahad Khalifa

**Affiliations:** 1 General Dentistry, Dental College, Ibn Sina National College for Medical Studies, Jeddah, SAU; 2 Orthodontics and Dentofacial Orthopaedics, King Fahad Military Medical Complex, Jeddah, SAU; 3 Periodontics, Faculty of Dentistry, King Abdulaziz University, Jeddah, SAU; 4 Periodontic Division, Specialized Dental Center, King Fahad Armed Forces Hospital, Jeddah, SAU

**Keywords:** interrelationship, interdisciplinary, forces, plaque accumulation, orthodontic treatment, periodontal treatment, clinical assessment, orthodontics, periodontics

## Abstract

Orthodontics and periodontics are intricately linked since adult orthodontics often implicate the periodontium. Periodontal intervention is needed throughout all phases of orthodontic treatment, from orthodontic diagnosis to mid-treatment periodontal assessment and postoperative evaluation. Invariably, periodontal health affects orthodontic success. Conversely, orthodontic tooth movements may serve as adjunctive therapy in patients with periodontal disease. This review aimed to provide a comprehensive understanding of the orthodontic-periodontic relationship for optimizing therapeutic strategies and achieving the best treatment outcomes in patients.

## Introduction and background

In contemporary dental practices, the increased emphasis on dentofacial esthetics is largely considered the norm, and orthodontic treatment for children and adults is more in demand than ever before. Advances in dental materials and orthodontic techniques, along with more esthetically pleasing fixed appliance choices, such as ceramic brackets and lingual appliances, have further encouraged adult patients to seek orthodontic treatment. For these patients, the patient’s orthodontic needs and their periodontal conditions are often intertwined, thus, necessitating a multi-disciplinary management approach. When treating these patients, in-depth knowledge of orthodontics and periodontics is imperative.

The interplay between orthodontics and periodontics is mutually advantageous. Tooth movement during orthodontic therapy is accomplished through bone remodeling of the alveolus. Since the periodontal ligament facilitates the bone remodeling response, tooth movement depends on the periodontal ligament, which in turn, relies on a healthy periodontium [1]. Consequently, periodontal treatment is essential throughout most phases of orthodontic therapy, beginning with the orthodontic diagnosis and continuing through to mid-treatment periodontal assessment and post-treatment evaluation. Common orthodontic issues typically seen in patients with compromised periodontal health include maxillary anterior teeth proclination, absence or loss of interdental spacing, rotated teeth, super-eruption, pathologic tooth migration, tooth loss, as well as traumatic occlusion. In contrast, orthodontic treatment may be warranted during periodontal treatment. For example, teeth may be orthodontically moved to facilitate oral hygiene and reduce bacterial loading and biofilm formation, correcting abnormal gingival and bone patterns, improving appearance, and aiding prosthetic replacement [2]. To enhance the overall treatment outcome, the periodontist and the orthodontist must collaboratively evaluate all periodontal situations and select the most appropriate orthodontic interventions.

This review aimed to present a comprehensive understanding of the orthodontic-periodontic relationship and discuss how the two disciplines can work together to improve patient care. 

## Review

 Methods

An electronic search was performed for all articles published on the orthodontic-periodontic challenges in integrated treatment in various databases including PubMed, SJR, Google Scholar, and Web of Science, from January 1, 1970, to December 31, 2023.

The inclusion criteria were as follows: 1) Articles written in English. 2) In-vivo studies that involved adult human male or female patients. 3) Full-text research. 4) Prospective or retrospective studies including randomized controlled trials, case series, and case reports. 5) Review papers. 6) Books. Moreover, the period of time for published articles between 1970 and 2023 were included.

The exclusion criteria were as follows: 1) Published studies in any language other than English. 2) Animal and in-vitro studies. 3) Research with irrelevant outcomes or outcomes unrelated to the fields of orthodontics/periodontics. 4) Journals that are not cited in the open access checklist for predatory publishers. 5) Published articles before January 1, 1970 were excluded.

Results

Articles were identified using the following search terms: periodontics, orthodontics, clinical assessment, periodontal treatment, orthodontic treatment, plaque accumulation, forces, interdisciplinary, and interrelationship.

**Figure 1:**
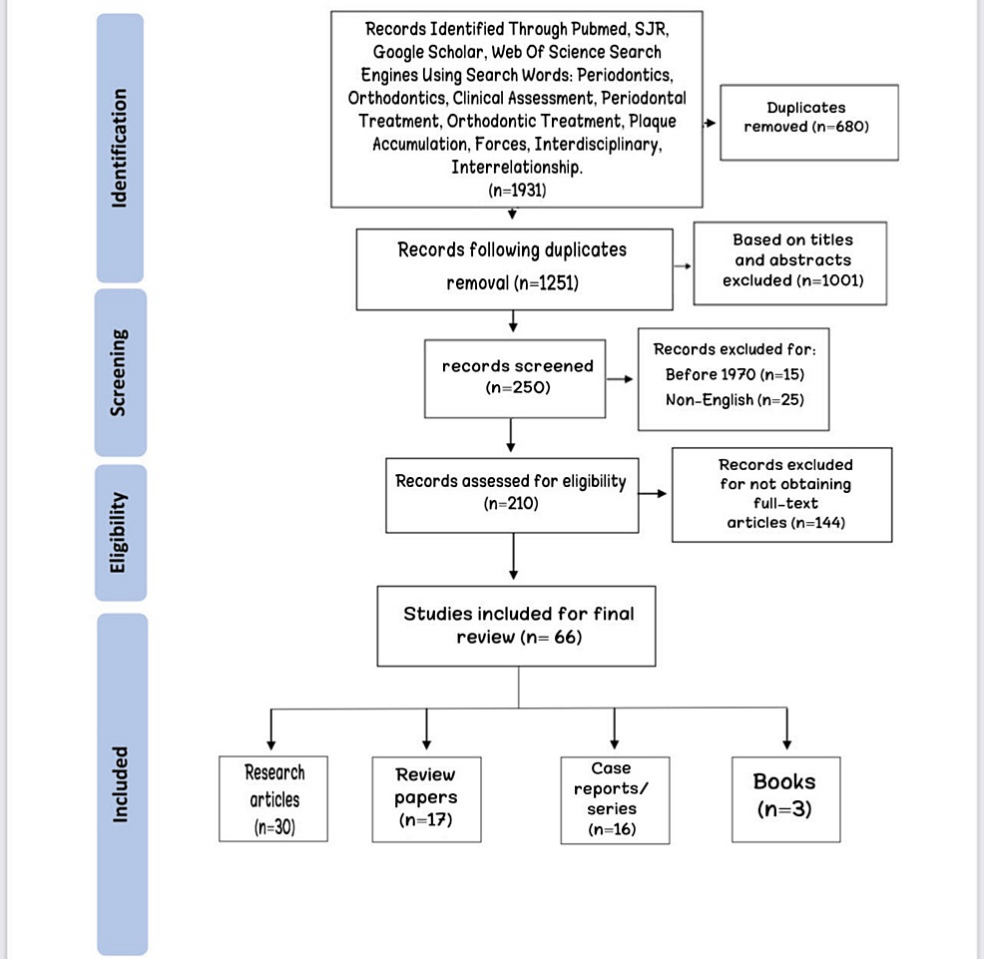
Flowchart describing the study design process.

During the initial search, 1931 articles were identified based on the keywords used. Of these, 680 articles were removed due to duplicates. In addition, 1001 articles were excluded based on the titles and abstracts. Then, 250 articles were deemed eligible for screening. Moreover, articles that were written before 1970, available in a language other than English, and for which the full text was not available, were also excluded. Finally, 66 articles were included in the analysis. These included papers were Research articles [n = 30], Review papers [n = 17], Case reports/series [n = 16], and Books [n = 3]. Figure 1 shows a flowchart of the literature search process.

 Discussion

 Orthodontic Treatment Effects on the Periodontal Parameters

 Oral Hygiene Maintenance

Studies have shown that if gingival inflammation is maintained under control, orthodontic therapy does not adversely affect periodontal attachments [3]. However, fixed orthodontic appliances can create barriers that compromise oral hygiene thus leading to plaque retention [4-5]. By compromising effective oral hygiene, orthodontics can be considered a risk factor for periodontal disease.

In the malaligned dentition, effective oral hygiene can be challenging due to the difficulty in reaching all the areas during toothbrushing and flossing, which can result in increased inflammation and bleeding [6]. Only a few clinical researchers have reported on periodontal disease and significant clinical attachment loss (CAL) in the maxilla and mandible in orthodontic patients. This may be due to poor oral hygiene in the molar regions due to the presence of molar bands, which can also lead to food impaction [7]. However, when a comprehensive dental care regimen is implemented during orthodontic therapy, adverse change, such as gingival bleeding [7-8] or dental plaque quantity [7-9], are limited. By facilitating plaque removal, a better periodontal condition is achieved. Gusmao [10]. investigated 90 patients with malocclusion and found that gingival recession was associated with teeth directed toward the cheeks or mouth, chronic periodontitis, excessively proclined maxillary incisors, dental crowding, and diastema.

Bracket Positions and Molar Band Placements

Subgingivally placed orthodontic bands may encroach on the alveolar bone. Banded appliances also impact periodontal health differently than bonded appliances. Banding, compared to bonding, is associated with increased inflammation and more severe attachment loss [11]. Gingival hyperplasia due to the presence of orthodontic bands, which may give rise to “pseudo periodontal pockets” [12]. However, the gingival hyperplasia generally resolves within a few weeks of disbanding. To minimize hyperplasia, it is essential to ensure that the slots on orthodontic bands are positioned perpendicularly to the tooth's long axis rather than being aligned with the aligned parallel to the occlusal plane [13]. Also, if brackets are placed relying on incisal edges, the increased root divergence could result in an open gingival embrasure that is unsightly [13]. For periodontal causes, orthodontic treatment using The Clear Aligner system appears to be significantly more beneficial in patients with intact periodontium than lingually or labially fixed appliances [14-15].

Orthodontic forces and periodontal health

When teeth are moved orthodontically, the forces applied to teeth are regulated forces. As a result of the applied forces, the dental and periodontal tissues undergo remodeling. The literature suggests that for a compromised periodontium, continuous light forces between 5 g and 15 g are preferable. Moreover, the time it takes for tooth mobility to begin after periodontal surgery ranges from seven days to one year [16]. Furthermore, several studies have revealed that the degree of root resorption increases as force magnitude increases [17-18]. Whether the presence of continuous or intermittent pressures is linked to an increased risk of root resorption is determined by the magnitude of the force. Many researchers agree that using intermittent forces generally leads to minimal root resorption because the disrupted cementum is given time to heal in between tooth movements [19-20].

Orthodontic treatment in supplement to periodontal therapy

In many cases, orthodontic therapy may be used in conjunction with periodontal treatment. Numerous orthodontic procedures, including the uprighting of teeth, intrusion, and rotation, are used to correct pathologically migrated teeth to arrest the progress of periodontal disease, and enhance the patient’s quality of life by improving oral function and appearance. Orthodontics should only be carried out if active periodontal disease has been successfully managed.

Teeth movement association with infra-bony defects

In the absence of inflammation and the presence of appropriate bacterial plaque control, orthodontic movement of teeth with infra-bony defects is possible. Despite limited evidence from clinical studies, tooth movement through an intra-bony defect is thought to occur by "carrying the bone" with the tooth, thereby improving the bone defect and positions of teeth close to an implant or replacement tooth [21].

Orthodontic extrusion

Gingival tissue remodeling has been found to follow tooth movements. Orthodontic extrusion, also known as “forced eruption,” involves the repositioning of the intact connective tissue attachment of a tooth in a more coronal position, followed by bone deposition [21]. This can be applied to increase the clinical crown height and/or change the height of the free gingival margin [22-23]. As clinical attachment is associated with the cementoenamel junction (CEJ), the crestal bone is approximately preserved. Consequently, the crestal bone associated with the extruded tooth and the adjacent teeth are increased (or remodeled), thereby, shallowing out the intraosseous deformities. In clinical crown lengthening, orthodontic extrusion with supra-gingival fibrotomy is suggested. However, osteotomy of the crestal bone is not indicated [24-26].

 Orthodontic extrusion may be a viable option for treating vertical periodontal defects [27]. For example, the dental extrusion of an irreversible tooth before implant placement seems to be an effective option for bone augmentation in the implant recipient site. In this case, the buccolingual thickness of the alveolar ridge should also be increased. The recommendation is to extrude the tooth progressively using constant low forces, ensuring that no more than 2 mm of labial root torque occurs every month. Furthermore, prior to tooth extraction, a retention duration of more than one month is desirable. Although similar to surgical augmentation, the overall therapy time involved in orthodontic extrusion may be shorter [28].

Orthodontic intrusion

Orthodontic tooth intrusion is performed in patients with maxillary anterior supraeruption and normal vertical maxillary development. It can also be indicated for tooth crown lengthening and treatment of teeth with horizontal bone defects or infrabony pockets. It is essential to keep these teeth in the intruded position for least 6 months to allow the principal periodontal fibers to adjust to the newly intruded position [29]. If oral hygiene has been maintained properly, the application of intrusive orthodontic forces may encourage favorable periodontal modifications [30]. In contrast, oral care is poor, intrusive movements may encourage the development of bacterial plaque in the subgingival region, thereby, exacerbating the continuing periodontal destruction process [31-32]. During active intrusion of a tooth, proficient supragingival and subgingival debridement is essential. When moving teeth, light forces, ranging from 5 g to 15 g per tooth, which may also decrease the risk of root resorption [2].

Furcation defects require extra and focused care during orthodontic treatment. In these cases, maintenance is a constant struggle and the furcation defects may worsen during orthodontic treatment. When dealing with a Class III furcation, hemisectioning the tooth and separating the roots may be more effective [33]. To restore a supraerupted tooth with an osseous defect, it may be necessary to intrusion the defect and then level it off.

Guided tissue regeneration (GTR) may be relevant in particular situations in association with orthodontic intrusion. However, there are currently no clear indications. According to published case reports, where orthodontic intrusion is required, implementing GTR procedures prior to orthodontic intrusion leads to new bone growth and gingival reattachment [34-35]. Other studies have reported that intrusion could enhance blood flow and deepen a specific bony defect, creating favorable conditions for GTR techniques [36-37].

Uprighting permanent molars

The upright of mesially inclined molars involves distal tooth movements, which enable the mesial defect to be filled with alveolar bone. A gingival overgrowth as well as plaque retentive region on the mesial side are also removed [38]. The mesial inclination of the second molar, particularly the lower one, caused by the absence of the first molar, is a common clinical finding. The risk of developing localized periodontitis is increased by this mesial inclination. Orthodontic uprighting is widely regarded as a beneficial treatment for reducing or eliminating existing periodontal problems. Orthodontic uprighting of mesially inclined teeth has not been associated with subsequent pocket development, improvement, or elimination [39-40].

Periodontal therapy as a supplement to orthodontic therapy

To reduce inflammation, periodontal treatment is recommended prior to orthodontic treatment. If there are any mild to moderate periodontal pockets present, phase 2 (surgical) periodontal treatment is initiated if better plaque control and bleeding on probing could be achieved, regardless of the orthodontic treatment. Even though pocket depths and bleeding on probing cannot be eliminated following initial periodontal treatment, open-flap debridement is still recommended as the first step [41].

Before proceeding with orthodontic tooth movement, regenerative periodontal therapy may be recommended in cases with shallow osseous crater-type bone defects (e.g., pocket depths of 4-5 mm). This can also minimize the need for surgical periodontal therapy during orthodontic treatment. However, the deep intra-bony defects [42], or deep osseous crater-type bone defects, may not improve with orthodontic treatment. Due to the unfavorable crown-root ratio and CAL after osseous resection, complete cleaning of deep periodontal defects is impossible. Of note, periodontal therapy must be completed 3-6 months before the initiation of orthodontic treatment [30, 43].

Proclination or labial tooth movements

The proclination of teeth, or labial tooth movements are the most practical way to treat dental crowding. These movements are frequently believed to be the cause of gingival recession [44-45].

 Two millimeters of keratinized gingiva are all that is needed for good periodontal health [46]. When orthodontic forces lead to tension on the gingival margin, gingival recession results [8]. The risk of recession and attachment loss is increased by orthodontic proclination of the incisors, particularly in areas where gingival and bone support are lacking, such as the lower incisor area. In such cases, mucogingival surgery may be recommended as part of orthodontic treatment to preserve the appropriate width of the attached gingiva [47]. It is assumed that there is no risk of recession if orthodontic movements occur within the genetically or functionally predetermined "bone envelope" [48]. Recently, a longitudinal study of adults with mandibular prognathism who underwent orthognathic surgery in 2008 by Ari-Demirkaya and Ilhan [49] demonstrated that such decompensation, which required extreme mandibular incisor labial tipping, did not lead to any negative effects on the periodontal structures. This study supports the 'bone envelope' hypothesis.

Although proclinations and labial tooth movements do not always result in gingival recession, in some cases, especially areas with a low resistance to inflammation or trauma, dehiscence of thin soft tissues or bones may result [50]. Importantly, lingual tooth movements can cause increased labiolingual gingival widths and slight incisal gingival migration, while labial movements cause the opposite.

Unequal gingival margins

In terms of overall esthetic appeal, the evenness of the gingival margin of the six maxillary anterior teeth is crucial [51]. For a balanced and attractive smile, the gingival margins of the maxillary central incisors should be set 1 mm below their CEJ and at the same level as the cuspid margins. Teeth adjacent to the lateral incisor may have a lower margin of 1-2 mm [52]. Variations in the gingival position or ectopic tooth eruption may lead to differences in the gingival margin level. There is no need to make any adjustments if the uneven gingival margin is not visible. If the shorter tooth has a deeper sulcus, an excisional gingivectomy may be performed to bring the gingival margin closer to the crown of the tooth. When the labiolingual thickness of one incisal edge is greater than that of the adjacent tooth, it may be an indication that the tooth has already overerupted or abraded. To correct these abnormalities, it is best to have orthodontic and periodontal treatment provided by the same interdisciplinary team. Light orthodontic forces should be used to intrude the overerupted teeth. To lower the risk of relapse, orthodontic intrusion should be completed six months before appliance removal [53].

The missing interdental papilla

Another crucial esthetic aspect of someone’s appearance is whether or not they have a papilla between their maxillary central incisors. Individuals missing gingival papillae or open gingival embrasures can be a challenge to treat with periodontal treatment. Orthodontic treatment, on the other hand, may assist. An open interdental space is generally caused by three aspects: the shape of the tooth, the angle of the root, or bone loss. To choose the most appropriate management, the following needs to be assessed: (1) the distance between the contact points as well as the bone crest, (2) the papillary height as in the interdental space, (3) the extent of root divergence, and (4) the tooth form. First, it is important to determine whether the tooth contacts or the papilla are responsible for the issue. If the papilla is the issue, the underlying periodontal problem is likely the cause of the lack of bone support. Additional treatment, such as enameloplasty (space reduction between teeth), tooth movement, and also the selective composite resin additions may be necessary [52]. A new papilla is formed by the soft tissues between the teeth being compressed after proximal recontouring, as well as orthodontic tooth approximation.

Numerous open embrasures have been induced by tooth contact issues. These should be assessed with a periapical radiograph of the central incisors. Radiographically, if the root angulations are divergent from one another, brackets may be used to adjust the root position. If roots are properly angled, an open contact could be due to an abnormal tooth shape, such as wide, triangular, or otherwise bell-shaped teeth. Black triangles can result when a point contact is close to the tooth's incisal edge [52]. In most cases, diastema closure and interproximal enamel reduction are sufficient to restore the absent papilla [54].

Excessive gingival display

In keeping with the current esthetic standards, the maxillary gingival display in an appealing individual smile should be around 1-2 mm. The extent of gingival display varies the greatest among children and gradually reduces with age [55]. Clinicians should always consider the patient's age when diagnosing and proposing treatment for gummy smiles [56]. Before starting orthodontic treatment, gummy smiles should be evaluated to determine possible etiology. For example, gummy smiles may be caused by a dynamic upper lip, an abnormally short lip, excessive lower facial height, a significant increase in the vertical dimension of the maxilla, extrusion of the anterior dental alveolus, or a shortened clinical crown. Treatment may be different depending on the etiology and must be handled properly [57].

A gummy smile can also be caused by vertical maxillary excess, in which dental crowns are frequently present with normal dimensions and healthy gingiva. In most cases, maxillary impaction and a LeForte I osteotomy are the treatment of choice in adults. For pediatric patients, "first bicuspid extractions preceded by applying a high pull J-hook headgear to the premaxillary segment" can limit the progression of this issue [58].

Certain patients occasionally experience significant delays in the gingival margins' physiological apical migration, together with probing depths of 3-4 mm in the gingival sulcus. A short clinical crown and a significantly increased labiolingual gingival tissue thickness are the two key clinical hallmarks of this type of gummy smile. Furthermore, if cosmetic concerns are not extreme, esthetic approaches to changing or repositioning the gingival marginal should be postponed and re-evaluated in early adulthood. During adolescence, there is a high probability that the gingival level will change, in both shape and position [59-60]. Extrusion of the maxillary front teeth typically causes an excessive gingival display. This is a common occurrence in Angle class 2 division 2 malocclusions. Maxillary incisor orthodontic intrusion is frequently advised as a treatment for these patients, which helps to eliminate a gummy smile [61].

Gummy smiles produced by a low upper lip philtrum can be treated neuromuscularly with Botox, which yields good but transitory results [62].

Orthodontics with corticotomy

Multiple forms of corticotomy-assisted orthodontics (CAO) have been used to speed up orthodontic treatment. Henry Köle developed corticotomy, a fast method of tooth movement, in 1959 [63]. It is widely accepted that the bone's cortical plates pose the greatest challenge to orthodontic tooth movements. In CAO, dental and marginal periodontal health are preserved, while a precise cut is made to disrupt the continuity of the cortical bone to accelerate tooth movement [63].

Three key operations comprise Köle's surgical method: 1) a vertical lingual incision, 2) a vertical buccal incision, and 3) a subapical horizontal osteotomy. CAO renders the targeted tooth and bone as an entire "bony block," and thus, advancing tooth movement more quickly. However, due to the high level of trauma and poor patient acceptance, this procedure has not been widely used.

The tissues regenerate more quickly than usual due to local tissue reactions to noxious stimulations (without corticotomy). As a result of increased osteoblastic-osteoclastic activities and elevated inflammatory mediator levels in the areas surrounding the cuts, bone turnover is accelerated and orthodontic tooth movements are quickened [64].

The CAO has many advantages over conventional tooth movements including shortened treatment time, simpler arch expansion, and a lower root resorption rate [64]. Additionally, it enhances post-orthodontic stability and reduces the chance of relapse [65].

In 1975, Düker [66] evaluated the CAO in a beagle hound and Köle refined his surgical technique by moving the locations of the buccal and lingual vertical incisions from two to three millimeters (mm) beneath the alveolar crest to just two millimeters (mm) below the apex.

What is now known as a corticotomy generally refers to a modified corticotomy, which includes a wide flap to allow for complete alveolar bone exposure and facilitate cuts among teeth, to reduce the forces required to move teeth [67]. A recent research found no evidence of relative negative impacts, such as resorption of the root surfaces or periodontal damage [68].

Periodontally accelerated osteogenic orthodontics

Periodontally accelerated osteogenic orthodontics (PAOO) is a rapid osteogenic orthodontic approach. It is also referred to as “Wilckodontics” as the technique was reported by Wilcko et al. in 2001. PAOO involves elevating full-thickness labial and lingual flaps, inserting bone grafts, closing the flaps surgically, and then, applying orthodontic forces to teeth. This treatment requires further labial and lingual cortical bone surgery (corticotomy) and may scar [65].

A modified PAOO technique was described by Wilcko et al. in 2009 [69], which reduces orthodontic treatment time by half or more. Results from other studies [68, 70-71] have also demonstrated that PAOO can lessen the time of orthodontic treatment, and increase bone thickness, and in some cases, orthognathic surgery can be avoided.

Later, Wilcko emphasized the regional acceleration phenomenon (RAP). RAP explains that the rapid tooth movements were caused by decortication and that alveolar surgical trauma may stimulate the reconstruction of surrounding tissues and more frequent remineralization and demineralization of the bone tissue [69]. Frost first suggested the RAP in 1983, and described it as a tissue reaction to nearby stimuli [72]. Orthodontic forces reposition teeth by relying on the mineralized and demineralized matrices of the bone tissue surrounding tooth roots to remodel. RAP operates on the basis of stimulating bone by cutting through the bony cortex of the alveolar bone, which accelerates local responses within the alveolar bone [73].

Regional acceleration phenomenon begins a few days after incisions to the cortex and peaks during the first and second month, persisting for four months, and then, gradually decreases between 6 and 24 months [74]. The RAP phenomenon, on the other hand, will continue as long as the teeth are still moving. When orthodontic tooth movements are accomplished, the effect will resolve, and the supporting alveolar bone will be remineralized [74-75].

One week before surgery, most components of the fixed orthodontic appliance (i.e., braces) should be inserted, with the archwires activated. In rapid tooth movement, the orthodontist has four to six months to complete the treatment. After four to six months, the pace of tooth movement returns to normal [73].

Below are a few examples of using PAOO in orthodontics:

 (a) Crowding, particularly moderate to severe crowding. (b) Canines that need to be retracted rapidly. (c) Exposure of impacted teeth to facilitate eruption. (d) Maxillary arch expansion. (e) Orthognathic surgery in which a shorter treatment time is desired [76-78].

Benefits of PAOO

(a) PAOO after surgery has greater stability than conventional orthodontic treatment. (b) It provides the least resistance to tooth movement where the bone has been removed, while the bone that has been preserved is less impacted by tooth movement overall. (c) Bone grafting increases the quantity of alveolar bone, and strengthens the periodontal tissue, which in turn, reduces the need for anchoring devices. (d) Root resorption is reduced, and anatomical variations are preserved. Several studies have suggested that PAOO decreases both the resistance to tooth movements and the force used during tooth movements [79-80].

Modern surgical methods

A further ultrasonic-assisted approach, known as piezopuncture, was examined by Kim et al. in 2013. In this method, ultrasonic surgical equipment is used to penetrate the bony cortex via the surrounding associated gingiva.

A clinical study by Alikhani [81-83]. examined the impact of micro-osteoperforations (MOPs) on the rate of tooth movement. The results showed that MOPs increased the speed of tooth movement by a rate of 2.3. In contrast, several systematic reviews [81, 84] have concluded that the MOPs approach does not speed up tooth movements and that there is inadequate evidence to support the use of MOPs alone for facilitating rapid tooth movements.

Piezocision-facilitated orthodontics

In 2020, Sivarajan et al. [85] created a cutting-edge minimally invasive surgical method namely piezosurgery. This method facilitates rapid tooth movements, without the need for protracted treatment or risky surgical procedures. Moreover, the tunnel technique and bone and soft tissue grafting are preserved in this method [85].

Piezosurgery appears to have additional advantages, such as being safer, less traumatic, and less invasive [86]. In certain circumstances, piezocision may be combined with Invisalign to reduce treatment time, while also meeting the patient’s expectation for an esthetic appliance [87]. The literature shows that, unlike traditional orthodontics, Piezosurgery method does not cause any further root resorption or periodontal trauma [87].

Our comprehensive review summarized and appraised all peer-reviewed studies published between 1970 and 2023 that fulfilled both our exclusion and inclusion criteria. To our knowledge, our study is the only in-depth comprehensive review that covered the topic of orthodontic-periodontics challenges in integrated treatment. In addition, we used SJR, Public Medline (PubMed), Web of Science, and Google Scholar as search engines. One feature of using Google Scholar is to prevent missing any relevant research published in journals that are still not indexed in PubMed. On the other hand, due to heterogeneity of the final selected articles (66 articles) with respect to the use of varied integrated treatments, we were restricted to performing a comprehensive review with different treatment options. This review excluded all the full-text articles with different languages except the English language that may increase the number of including studies, if the inclusion and exclusion criteria were included the other non-English languages with full-text article. In addition, although research articles conducted in human subjects were part of the inclusion criteria, unfortunately, up to date, we found most of the published studies were in vitro studies or animal studies that evaluated the orthodontics-periodontics challenges in integrated treatment.

A challenge of orthodontics-periodontics using integrated treatment that have not been explored are now clear and should be investigated to better understand these challenges of strength and limitations before going to future clinical trials. These challenges may include oral hygiene maintenance, teeth movement association with infra-bony defects, orthodontic extrusion/intrusion in addition to other untested periodontal diseases. Understanding the causes of orthodontic-periodontics challenges are very important part of integrated treatments and may help sensitize many patients. As a result, dental professionals in orthodontics and periodontics should emphasize and engage their dental society in change the traditional treatment options against orthodontics-periodontics diseases. We are thinking the international dental association conferences a great opportunity to encourage the young specialties in orthodontics and periodontics from different countries to recognize the suitable treatment consequences of orthodontics-periodontics diseases toward their dental societies, through community-based awareness campaigns. More active participation of academical/educational institutions is needed to further reduce the orthodontics-periodontics challenges in integrated treatment of orthodontics-periodontics diseases over all the world. Future longitudinal randomized clinical trials are required in this orthodontics-periodontics field.

## Conclusions

Orthodontics and periodontal health are intimately associated. On the one hand, orthodontics may eliminate areas that retain plaque. On the other hand, a dynamic periodontium is essential in facilitating orthodontic tooth movements. An increasing number of adults are considering orthodontic treatment as a result of changing lifestyles and aspirations. In these circumstances, an integrated orthodontics-periodontics approach is helpful and can contribute to ideal qualitative, functional, as well as esthetic planning, leading to optimized treatment plans, especially in complex clinical cases. Furthermore, new periodontal surgical techniques, such as PAOO and piezocision, may enhance orthodontic tooth movements, leading to decreased treatment timeframes, while simultaneously boosting treatment effectiveness.
